# The impact of dental caries and its treatment under general anaesthetic on children and their families

**DOI:** 10.1007/s40368-020-00591-1

**Published:** 2020-12-05

**Authors:** R. Knapp, Z. Marshman, F. Gilchrist, H. Rodd

**Affiliations:** School of Clinical Dentistry, University of Sheffield, Sheffield, UK

**Keywords:** Oral health, Quality of life, Paediatric dentistry, Caries

## Abstract

**Objective:**

To assess the impact of dental caries and treatment under general anaesthetic (GA) on the everyday lives of children and their families, using child-reported measures of quality of life (QoL) and oral health-related quality of life (OHRQoL).

**Method:**

Participants, aged 5–16 years old having treatment for dental caries under GA, were recruited from new patient clinics at Charles Clifford Dental Hospital, Sheffield. OHRQoL was measured before and 3-months after treatment using the Caries Impacts and Experiences Questionnaire for Children (CARIES-QC). Overall QoL was measured using the Child Health Utility 9D (CHU9D). Parents/caregivers completed the Family Impact Scale (FIS).

**Results:**

Eighty five parent–child dyads completed the study. There was statistically significant improvement in OHRQoL (mean interval score difference in CARIES-QC = 4.43, *p* < 0.001) and QoL (mean score difference in CHU9D = 2.48, *p* < 0.001) following treatment, with moderate to large effect sizes. There was statistically significant improvement in FIS scores (mean score difference = 5.48, *p* = 0.03).

**Conclusions:**

Treatment under GA was associated with improvement in QoL and OHRQoL as reported by children, and reduced impacts on the family. This work highlights the importance of GA services in reducing the caries-related impacts experienced by children. Further work is needed investigate the impact of clinical, environmental and individual factors.

## Background

Dental caries remains one of the most prevalent chronic diseases of childhood, affecting 60–90% of children worldwide. There is wide variation between with within countries, for example, in Europe between 20 and 90% of children aged six have dental caries (Petersen 2003). Although overall rates of dental caries are decreasing, these figures have shown inequalities within countries and across the region, with the highest burden of disease carried by those from lower socioeconomic groups (Petersen et al. 2005; Jakab 2016). In the UK, the most recent child dental health survey reported 31% of 5-year-old children had ‘obvious decay experience’, rising to nearly half by age eight (Steele et al. 2015). Many children with dental caries receive treatment under general anaesthetic (GA) and in the UK, it remains the most common reason for a child to be admitted to hospital. In 2018/2019, in England alone, there were approximately 59,000 ‘Finished Consultant Episodes’ for children and adolescents (aged 0–19) admitted for dental extractions under GA (Public Health England 2020). These statistics are shocking when we consider that dental caries is an almost entirely preventable disease.

While impacts of caries are well documented, what is less well understood is the subjective experience of children themselves. Several instruments have been developed to investigate the subjective impact of oral diseases, seeking to measure oral health-related quality of life (OHRQoL). A number of studies have demonstrated overall improvements in OHRQoL in children following treatment for dental caries under GA, however, most studies of child-OHRQoL have relied on proxy-reported measures rather than seeking the views of children themselves (Knapp et al. 2017). In addition, earlier studies have relied on generic measures of OHRQoL to measure change following treatment, may not have been sensitive enough to capture caries-specific impacts (Guyatt et al. 1993).

Therefore, the aim of this study was to assess the impact of dental caries and treatment under GA on the everyday lives of children and their families, using child-reported measures of OHRQoL and overall quality of life (QoL).

## Materials and methods

Approval for this project was obtained from the South East Scotland NHS Research Ethics Committee (ref: 16/SS/0187). A convenience sample of participants was recruited from new patient clinics on the Paediatric Clinic, Charles Clifford Dental Hospital, Sheffield, UK between January 2017 and January 2019. A sample size calculation revealed that 84 participants would be needed to detect an effect size of 0.4 (i.e., medium effect) at 5% level of significance and 95% power.

All children, and their parents, who met the eligibility criteria for the study, were invited to participate. Inclusion criteria were: children aged 5–16 years; with active dental caries; who are otherwise medically fit and well; children and parents both able to understand spoken English, i.e., able to understand and undertake the research with support. Potential participants were excluded if they had caries in conjunction with any other dental conditions, e.g., trauma or dental anomaly, such as molar incisor hypomineralisation, as due to the numbers of participants involved there would not be sufficient data to account for this as a potential confounding factor in the results.

This study employed a pre-test/post-test design. Children were given age-appropriate information sheets at their assessment visit and, if they wished to participate, were asked to complete consent forms and questionnaires at the following time points: pre-test at their new patient appointment, and post-test, 3 months following treatment under GA, self-completed at home and returned by post. Children were asked to complete the questionnaire themselves, but younger children were given assistance if needed, e.g., having the questions read to them, but with no additional guidance on the answers. Parents were asked to complete a separate consent form and questionnaire at the same time points. Once participants had completed the study, they were given a £10 ‘Love to Shop’ gift voucher as a thank you for their time. Where parents did not agree to participate, the parent/child dyad was excluded from the study.

### Measures used

The child questionnaire consisted of two sections: the Caries Impacts and Experiences Questionnaire for Children (CARIES-QC) designed to measure OHRQoL and the Child Health Utility 9D (CHU9D) which measures overall QoL.

CARIES-QC had previously been validated for use in this population. It is a caries-specific questionnaire and was designed for completion by children themselves. It contains 12 items with a 3-point response format, where children rate whether they are affected ‘not at all’, ‘a bit’ or ‘a lot’, with respective scores of 0, 1 and 2. A global question asks children to rate how much of a problem their teeth are overall, on the same 3-point scale. Total raw scores range from 0 to 24, where higher scores indicate worse OHRQoL. To calculate change following treatment, raw scores are converted to an interval scale score, which allows for the more accurate calculation of change at all points on the scale (Gilchrist et al. 2018).

The CHU-9D consists of nine items, each with five ordinal responses (scored 1–5) that assess the child’s functioning across domains, such as worry, pain, tiredness, and daily routine. Overall scores range from 9 to 45, where increasing score implies greater impact on QoL (Stevens and Ratcliffe 2012).

The parent/caregiver questionnaire consisted of the Family Impact Scale (FIS) and additional questions regarding the child’s dental history. The FIS consists of 14 items, with 5-point Likert scale to record the frequency of each impact scored as follows: ‘Never’ = 0; ‘once or twice’ = 1; ‘sometimes’ = 2; ‘often’ = 3; ‘every day or almost every day’ = 4. A ‘don’t know’ response is also possible and scored as 0. This instrument is validated for use in this population (de Souza et al. 2016). Total FIS score ranges from 0 to 56, with higher scores representing greater family impact. Subscale scores were obtained by summing subsets of items within the categories of parental/family activity, parental emotions and family conflict. Global transition questions were also included. In terms of additional questions, parents were asked if the child had received antibiotics for their dental problem, and if so, how many courses, as well as whether the child or a sibling had received a dental GA previously. Information was obtained by parents in the questionnaire as it was not always available from the referral letter or patient record.

Demographic and clinical data were also collected, from the patient record. Individual characteristics included the age of the child, in years, and their ethnicity, recorded as ‘White British’ or ‘Black and Minority Ethnic’ (BME). Deprivation, which was assessed using a composite measure of area-based deprivation, the Index of Multiple Deprivation 2015 (IMD) score derived from their house postcode, was recorded. Both the overall IMD rank score and quintile were recorded, with quintile 1 being the most advantaged, and quintile 5 being the most disadvantaged quintile. The number of carious teeth was recorded based on clinical examination at their new patient appointment. This usually included caries recorded following radiographic assessment, therefore early carious lesions as well as cavitated lesions, unless the child was unable to tolerate radiographs being taken. Data were also collected on whether the child had anterior caries and whether they reported pain at the assessment, as well as if there were any safeguarding concerns in place. Safeguarding concerns were indicated in the patient record by the child’s placement on a historic or current care protection plan, whether they had a paediatric liaison letter or whether there was social care involvement (a named social worker, as detailed in the new patient proforma).

### Data analysis

Data were entered into an electronic database (IBM SPSS Statistics, version 24) and descriptive statistics reported. All statistical analysis results were considered significant at *p* < 0.05.

### Change following treatment

Total child-OHRQoL (CARIES-QC) and QoL (CHU-9D) scores at baseline and 3 months following treatment were calculated and change in scores analysed using the Mann–Whitney test. Change scores were calculated by subtracting follow-up scores from baseline scores, so a positive change indicated improvement in QoL. Cohen’s *d* effect sizes were calculated for change scores to assess the magnitude of change, with effect sizes of 0.2, 0.2–0.7 and greater than 0.7 representing small, moderate and large magnitudes of change, respectively (Sawilowsky 2009).

The minimal important difference (MID), i.e., the smallest difference in the score which is considered clinically meaningful and which patients perceive as beneficial (Masood et al. 2014), of the CARIES-QC and FIS scores, was calculated using the mean change scores of participants who reported ‘improvement’ on the global rating.

### Psychometric properties

Internal consistency was assessed using the Cronbach’s alpha test. By convention, the alpha cut-off value of 0.70 or higher was considered acceptable (Kline 1999). The longitudinal construct validity and responsiveness of CARIES-QC and FIS were assessed by comparing mean change scores for each measure with the global transition question responses. As CHU9D did not include a global question, it was not tested at this point.

## Results

### Sample characteristics

A total of 273 patients were approached to participate in the study between January 2017 and January 2019, of which 106 declined to participate (response rate = 61.2%). Of the 167 who consented to take part, 82 participants were subsequently lost to follow-up. In total, therefore, 85 parent–child dyads completed the study (completion rate = 50.9%). The age of children who completed the study ranged from 5 to 11 years (mea*n* = 6.5 ± 1.5). Nearly three-quarters (*n* = 62, 72.9%) of children were from the most deprived areas of England. Most children were White British (*n* = 62, 72.9%). There was no significant difference in these demographic variables between those who completed the study and those who did not (Table 1).

**Table 1:** Comparison of participants’ demographic characteristics at baseline, of those followed up and those lost to follow-up

Variable	All (*n* = 167)	Followed up (*n* = 85)	Lost to follow-up (*n* = 82)	*p*-value
Age, years				
Range	5–14	5–11	5–14	0.14
Mean (± SD)	6.70 (± 1.69)	6.49 (± 1.53)	6.91 (± 1.83)	
Gender				
Male	79 (47.3%)	38 (44.7%)	41 (50.0%)	0.49
Female	88 (52.7%)	47 (55.3%)	41 (50.0%)	
Ethnicity				
White British	121 (72.5%)	62 (72.9%)	59 (72.0%)	0.89
BME	46 (27.5%)	23 (27.1%)	23 (28.0%)	
Deprivation (based on IMD score)				
Least deprived	10 (6.0%)	6 (7.1%)	4 (4.9%)	0.46
Less deprived	19 (11.4%)	10 (11.8%)	9 (11.0%)	
Average	16 (9.6%)	7 (8.2%)	9 (11.0%)	
More deprived	36 (21.5%)	14 (16.5%)	22 (26.8%)	
Most deprived	86 (51.5%)	48 (56.5%)	38 (46.3%)	
Safeguarding concern				
No	138 (82.6%)	77 (90.6%)	61 (74.4%)	0.19
Yes	20 (12.0%)	8 (9.4%)	12 (14.6%)	
Data missing	9 (5.4%)	0	9 (11.0%)	

Numbers, with percentages in brackets, are given unless otherwise stated

*SD* standard deviation; *p*−values are for comparisons between the followed−up and lost to follow−up groups. As the data were not normally distributed, the Mann–Whitney *U* test was used to test for significant difference between the groups. Pearson’s Chi−squared test was used to test for difference in categorical variables. There were no statistically significant results. *BME* black or minority ethnic group

Clinical data from the patient record showed that the number of carious teeth ranged from 1 to 15 (mean = 6.6 ± 2.9). Anterior caries was present in 12% (*n* = 10) of the participants. Pain was reported at initial assessment by 70.6% (*n* = 60) of children. In addition, 8 (9.4%) children had safeguarding concerns in place. Antibiotics had been received by 43.5% (*n* = 37) of children prior to their assessment visit. Over a quarter (27%, *n* = 23) of children had a sibling who had previously received treatment under GA, while 2.4% (*n* = 2) of children had previously had dental treatment under GA themselves.

### Quality-of-life results

The total scores for each measure, before and three months after treatment, are given in Table 2. Effect sizes for the change in score are also given. There was a statistically significant improvement in OHRQoL (mean score difference in CARIES-QC = 4.43 ± 4.92, *p* < 0.001) and QoL (mean score difference in CHU9D = 2.48 ± 5.29, *p* < 0.001) following treatment, with moderate to large effect sizes.

**Table 2:** Mean overall scores at baseline and follow-up, with effect sizes

Measure	Baseline	Follow-up	Change	*p*-value	Cohen’s d effect size	Effect size description
CARIES-QC interval	8.99 ± 4.29 (0–19.96)	4.47 ± 5.58 (0–16.17)	4.43 ± 4.92 (− 8.63–16.92)	< 0.001*	0.91	Large
CHU9D	13.58 ± 4.96 (9–31)	11.09 ± 3.07 (9–24)	2.48 ± 5.29 (− 14–20)	< 0.001*	0.60	Moderate
FIS	9.21 ± 7.31 (0–35)	7.02 ± 6.40 (0–28)	2.19 ± 7.84 (− 17–24)	0.03*	0.32	Moderate

Scores show mean ± SD (range). *p*−values are for Wilcoxon test for difference between baseline and follow−up scores

*Statistically significant result (*p*<0.05)

At baseline, the main OHRQoL impacts reported by children were food getting stuck in their teeth (91%), having to eat on one side (64%) and their teeth causing them to cry (56%). All these impacts reduced following treatment under GA (Table 3).

**Table 3:** Number and proportion of children responding positively (‘a bit’ or ‘a lot’) to each item at baseline and follow-up

Item	Number (%) at baseline	Number (%) at 3-months follow-up	Reduction in % of children reporting this item at follow-up
Food stuck	73 (85.9%)	50 (58.8%)	31.5%
Feel cross	61 (71.8%)	13 (15.3%)	78.7%
Cried	61 (71.8%)	27 (31.8%)	55.7%
Eat more carefully	52 (61.2%)	22 (25.9%)	60.0%
Eating on one side	50 (58.8%)	32 (37.6%)	36.0%
Teeth hurt	48 (56.5%)	15 (17.6%)	68.8%
Hard to eat some foods	47 (55.3%)	27 (31.8%)	42.6%
Annoyed	45 (52.9%)	19 (22.4%)	57.8%
Eat more slowly	35 (41.2%)	17 (20.0%)	51.4%
Kept awake	33 (38.8%)	3 (3.5%)	91.0%
Hurt when brushing	33 (38.8%)	14 (16.5%)	57.6%
Hard to do schoolwork	17 (20.0%)	3 (3.5%)	82.4%

Data from the CARIES-QC global question revealed that, before treatment, 58% of the children said their teeth were a problem for them, and this figure reduced to 21% three months following treatment. Overall, 92% of children reported that their teeth were ‘better’ following treatment. The remaining 8% reported their teeth were ‘the same’ as before treatment.

The most common impacts on the family of these children before treatment were parents feeling upset (75.3%, *n* = 64), feeling guilty (75.3%, *n* = 63), having disturbed sleep (58.8%, *n* = 50) and the child requiring more attention (48.2%, *n* = 41). Nearly half the parents had to take time off work (45.9%, *n* = 39). Following treatment, the numbers of parents reporting each of these impacts reduced, albeit to different degrees. The biggest effects were seen in fewer parents having sleep disrupted and fewer feeling upset and guilty. There was a statistically significant change in overall FIS score following treatment (mean score difference = 2.19 ± 7.84, *p* = 0.03), however, only the ‘parental and family activities’ domain saw a statistically significant change in score between baseline and follow-up.

Responses to the global questions revealed that nearly all the parents (95%, *n* = 81) rated their child’s oral health as improved at follow-up. Approximately three-quarters of parents felt the overall quality of life of their children had improved (74%, *n* = 63), and none felt it had worsened. Just over half the parents (*n* = 46) reported that the impact on the family had improved, with the remaining parents reporting it had stayed the same.

The minimally important difference was 4.68 for CARIES-QC interval score and 1.65 for FIS. Overall, therefore, 40 participants (47.1%) exceeded the MID for CARIES-QC and 24 (28.2%) did so for the FIS.

### Psychometric properties of the measures used

CARIES-QC demonstrated excellent internal consistency (*α* = 0.9), CHU-9D and FIS both demonstrated good internal consistency (*α* = 0.8) overall.

Table 4 shows the results of longitudinal validity and responsiveness testing. CARIES-QC demonstrated good longitudinal validity and responsiveness overall; however, FIS demonstrated poor longitudinal validity and responsiveness.

**Table 4:** Mean change in quality of life scores by response to the global transition question

Response to GTJ	CARIES-QC Interval			FIS		
	Number (%)	Change score	*p*-value	Number (%)	Change score	*p*-value
Improved	78 (91.8%)	4.68 ± 4.81 (− 7.8–16.92)	< 0.001*	46 (54.1%)	1.65 ± 9.23 (− 17–24)	0.46
Stayed the same	7 (8.2%)	1.69 ± 5.61 (− 8.63–6.69)	0.49	39 (45.9%)	2.82 ± 5.83 (− 7–20)	0.009
Got worse	0	n/a	n/a	0	n/a	n/a

CARIES−QC interval scores show mean ± SD (range). *p*−values are for Wilcoxon test for difference between baseline and follow−up scores for each group

*Statistically significant result between scores at baseline and follow−up (*p*<0.05)

## Discussion

This study aimed to investigate the effect of treatment for dental caries under GA on the everyday lives of children and their families. In contrast to previous studies, CARIES-QC has provided insights into the aspects of a child’s daily life which are most improved following treatment, from their own perspective rather than relying on a proxy report. The other advantage to the use of CARIES-QC in this study, as the only disease-specific measure of OHRQoL available, is that it may have identified impacts which are specific to dental caries.

The results from this study have demonstrated that dental caries has a significant impact on children and their families. The main impacts of caries reported by children were related to eating and their teeth causing them to cry. The study has also demonstrated wider effects on the family and society, for example, parents feeling guilty and days lost at work. The findings, in relation to the sample characteristics and quality-of-life impacts are discussed in more detail below.

### Sample characteristics

The participants in this study had high levels of caries experience. The most recent survey of 5-year-old children in England found that the mean dmft for Sheffield children who had caries experience was 3.5 (Public Health England 2017). The dmft of children included in this study was considerably higher than this at 6.9, although the figure is similar to that in other studies investigating the impact of treatment under GA in children (e.g., Gilchrist et al. 2018).

This study clearly highlighted ongoing inequalities in children’s oral health as nearly three-quarters of those requiring treatment under GA in this sample were living in the most deprived areas of England. These findings support those in other studies where children receiving treatment under GA tended to be from socially deprived backgrounds (Hariharan et al. 2017). An important finding in this study was that over a quarter of children (27%) had a sibling who had previously received dental treatment under GA, highlighting the need for targeted oral health promotion and caries prevention programmes to help reduce inequalities in the burden of disease in deprived populations. Nationwide interventions have been used elsewhere in the UK with success in reducing inequalities, for example, ‘Childsmile’ in Scotland (McMahon et al. 2011) and ‘Designed to Smile’ in Wales (Morgan 2018). There is a need also to ensure there is sufficient remuneration for dentists to carry out preventative activity and to provide restorative care (Watt et al. 2019).

Additionally, there were several children in this study (9%, *n* = 8) who had safeguarding concerns in place. Perhaps more worryingly, a greater number (15%, *n* = 12) of children with safeguarding concerns in place were lost to follow-up, some of whom will have done so because they failed to return for further treatment. These children would have been processed through the departmental protocol for children who are not brought to appointments, which would have included communication with the safeguarding teams to ensure they were seen again at a later stage. However, this process means that there would have been a delay in these children receiving the treatment they required. Failure or delay in seeking dental care, including for dental caries, is a cause for concern, all the more so in the UK where child dental care is available free-of-charge on the NHS and cost is not a barrier to access. It is only more recently that missed appointments for dentistry have been considered from a safeguarding perspective (Harris 2018). This study highlights the impacts of dental caries which will be experienced by children who have a delay in receiving treatment under GA, either from not being brought to appointments, or where there is a lack of GA service provision resulting in long waiting times for treatment, or where GA services are reduced for other reasons, as has been the case during the recent COVID-19 pandemic.

The results from the study also show that significant numbers of children (43.5%) had received antibiotics prior to their initial assessment for a GA. These findings are concerning in the light of growing antibiotic resistance. It brings into question the prescribing practices of dentists, and whether these antibiotics are always indicated. It may be that antibiotics are viewed as a course of ‘treatment’ in the interim period from referral to assessment, where a dentist is unable to carry out treatment in the dental chair. Indeed, a survey of prescribing practices in the North of England found that there was only evidence of spreading infection or systemic involvement in approximately half of the cases, and that other reasons, such as patient expectations, time pressure and lack of co-operation, were impacting on the decision to prescribe antimicrobials (Sturrock et al. 2018). Although antibiotic prescribing in dentistry has reduced in recent years, 5.2% of all antibiotics are prescribed in dentistry (Public Health England, 2018), suggesting more needs to be done. Common approaches with medical colleagues to educate the wider public about the need to reduce antibiotic use and why antibiotics might not be appropriate is advocated, alongside support for dentists to have the confidence and time to do the right thing. This has implications for practice, such as ensuring urgent care appointments are available and appropriately remunerated to allow time for appropriate treatment to be carried out.

### Quality-of-life findings

There were statistically significant improvements in overall child OHRQoL scores (*p* < 0.001), child- HRQoL scores (*p* < 0.001) and in FIS scores (*p* = 0.03), 3 months following treatment under GA. The change in scores represented moderate to large effect sizes. All individual impacts were reduced following treatment, albeit to varying degrees. These results suggest that treatment under GA results in significant improvements in the OHRQoL and overall QoL of children, three months following treatment. The negative impact on the everyday lives of the families of these children was also significantly reduced following treatment.

CARIES-QC demonstrated good overall internal consistency (Cronbach alpha = 0.9). This value is slightly higher than that found in work by Foster-Page and colleagues (2019), who obtained an alpha of 0.8; and adds to the evidence that CARIE-QC has good overall internal consistency. CARIES-QC also good demonstrated longitudinal construct validity and responsiveness, supporting its use to evaluate change following treatment for dental caries in children. Interestingly, the large effect sizes seen for change in CARIES-QC scores in this study were greater than those previously reported in the study by Foster-Page and team (2019), who found moderate effect sizes. This may be because all the child participants in this study were requiring treatment under GA, which may reflect a greater treatment need than in the other study populations. In contrast, the FIS demonstrated poor validity and responsiveness in this population, and variable internal consistency. This may be because it is a generic rather than caries-specific measure, unable to detect changes in caries-related impacts on the family. Further psychometric testing of all measures, especially in larger samples is warranted. Factor and Rasch analysis may be helpful to establish which questions may not be performing as well.

### Limitations

It is worth noting some limitations of this study. While the completion rate of 50.9% in this study was comparable to similar studies (Martins-Junior et al. 2017)^,^ there was still a significant loss to follow-up. Comparison between those lost to follow-up and those completing the study confirmed that there was no difference in baseline characteristics between the two groups; however, future work should explore ways of trying to improve retention to such studies. It is well documented that individuals from low-income families are less likely to participate in research studies than those from higher-income backgrounds (Heinrichs et al. 2005). While the reasons for this are complex, this work highlights the need to consider how best to reach this group of patients. The inconvenience of completing and returning a paper questionnaire could be considerable. Although participants were given a gift voucher to try and compensate them for their time, it may have been that this was not seen as sufficient. A systematic review of strategies to improve retention in clinical trials found that the most effective method for increasing response rates to postal questionnaires was to give a monetary incentive, and the higher the incentive the higher the response rates (Brueton et al. 2014). It might be possible in future to make use of other methods, for example, by including other participatory approaches, such as drawings or activities, to encourage the involvement of children (Marshman and Hall 2008). In addition, children were only able to participate if their parents consented too, reducing the number of child participants. If possible, future work should consider child consent practices and the possibility of allowing children to participate even if their parents do not wish to.

One of the exclusion criteria was to exclude those with other dental conditions in conjunction with caries, as it was felt this could impact on OHRQoL scores. Ideally, future studies would seek to recruit all individuals with caries, but recruit in sufficient numbers that analysis could be made to determine if these additional dental conditions had a significant effect on OHRQoL outcomes.

Another limitation was the requirement for individuals to understand spoken English to allow completion of the questionnaire with support. This decision was made as, at the time of commencing the study, CARIES-QC was only validated for use in an English-speaking population. Translation may have altered the meaning of the questions and a translator may not have always been available. This could have led to selection bias in the sample, and therefore future work should seek to overcome this language restriction.

In conclusion, this research contributes to the field as it includes a disease-specific, child-reported measure to examine changes in OHRQoL following treatment for caries under GA. It has demonstrated that dental treatment under GA is associated with significant improvement in the OHRQoL and QoL of children, and in the impact on their families. Future work should be carried out to investigate these findings in more detail, taking into account individual, clinical and environmental factors which may impact on the results. The study has added to the evidence which shows all three measures demonstrate good internal consistency. This research also provided evidence of the usefulness of CARIES-QC but raised some questions regarding the usefulness of the FIS, in longitudinal research in similar populations.

## Conclusion

Treatment under GA was associated with significant improvement in QoL and OHRQoL as reported by both children and their parents. This work highlights the importance of dental care under GA for children with dental caries, which has implications for service planning and resource allocation. Treatment under GA requires significant theatre time and associated resources for treating dental caries in children, and this paper provides further evidence for both the impacts of caries on children and their families prior to treatment and the effectiveness of treatment in reducing those impacts. Further work is needed investigate the impact of clinical, environmental and individual factors on quality-of-life outcomes.
